# Supplementary figures and images for: Octopus track chemosensory plumes to find food
Source: PLoS One. 2025 Oct 8;20(10):e0330262. doi: 10.1371/journal.pone.0330262 (PMC12507230; doi:10.1371/journal.pone.0330262)

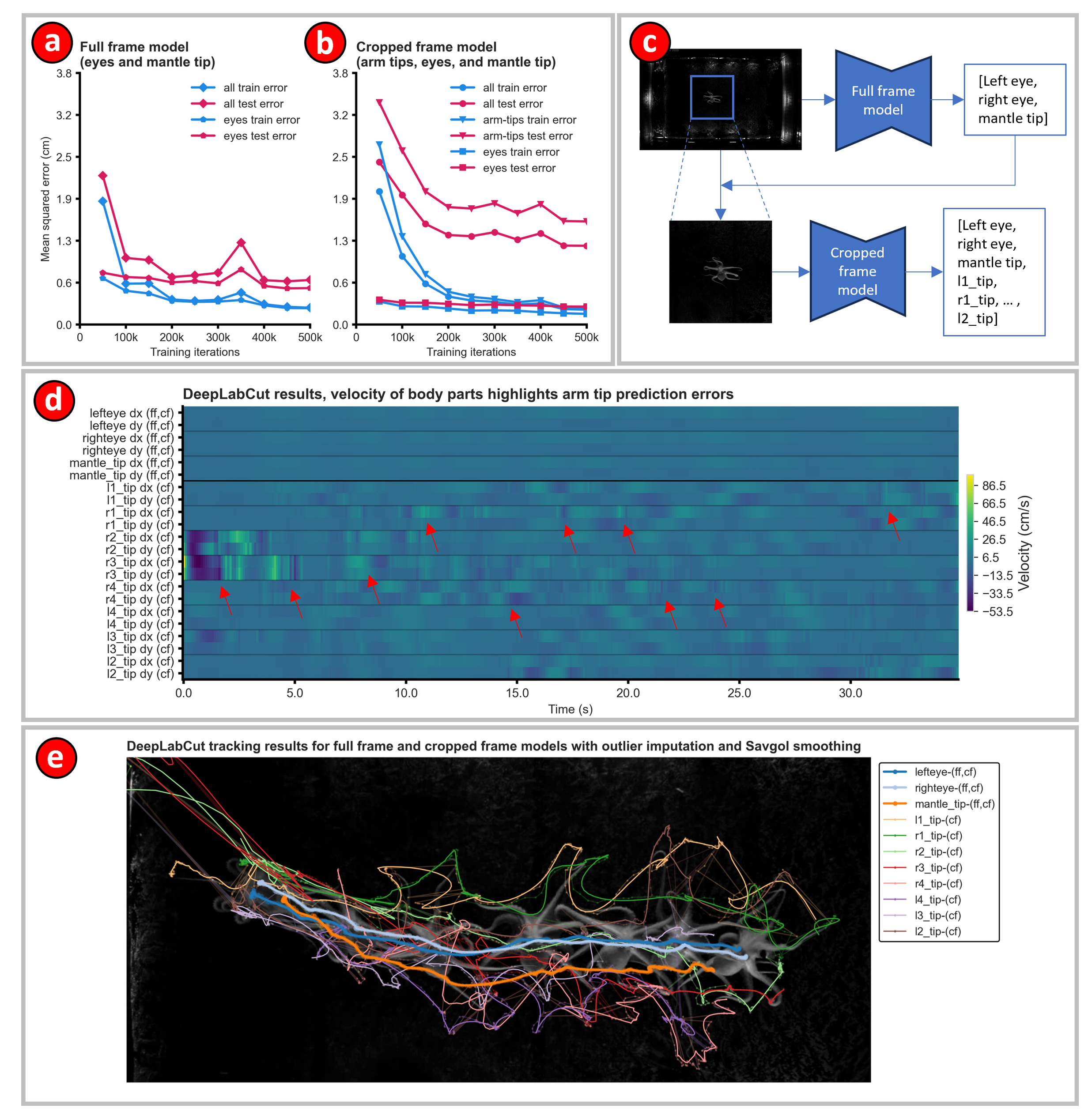

Supplement: S1 Fig — (a) Performance of the full frame model on tracking the eyes of the octopus. Eye tracking mean squared error test performance plateaued at 6 mm, a size smaller than the eyes of the octopus and on par with human error. (b) Performance of the cropped-frame model on tracking the eyes and arms of octopus Arm tip tracking mean squared error plateaued at 18 mm, a size far larger than the arm tips. Cropped frames were passed to DeepLabCut for arm tip annotations for two reasons, (1) the arm tip feature size is very small and the full frame resolution is large (1920x1200), and (2) humans find it much easier to annotate arm tips in cropped frames. (c) Flow chart of image processing sequence. (d) Visualizing body part velocities highlights the poor quality of arm tip tracking Velocity traces for each of the arm tips were thresholded to remove outliers and then smoothed using a Savitsky-Golay filter. Despite this the data showed large (both positive and negative), unphysical values for the arm tip velocity (red arrows) due to tracking errors. (e) DeepLabCut traces for all body parts for an example chemosensory tracking sequence. Solid thick lines are the smoothed traces while the thin faint lines of the same color are the raw traces. (TIF) [file pone.0330262.s001.tif]

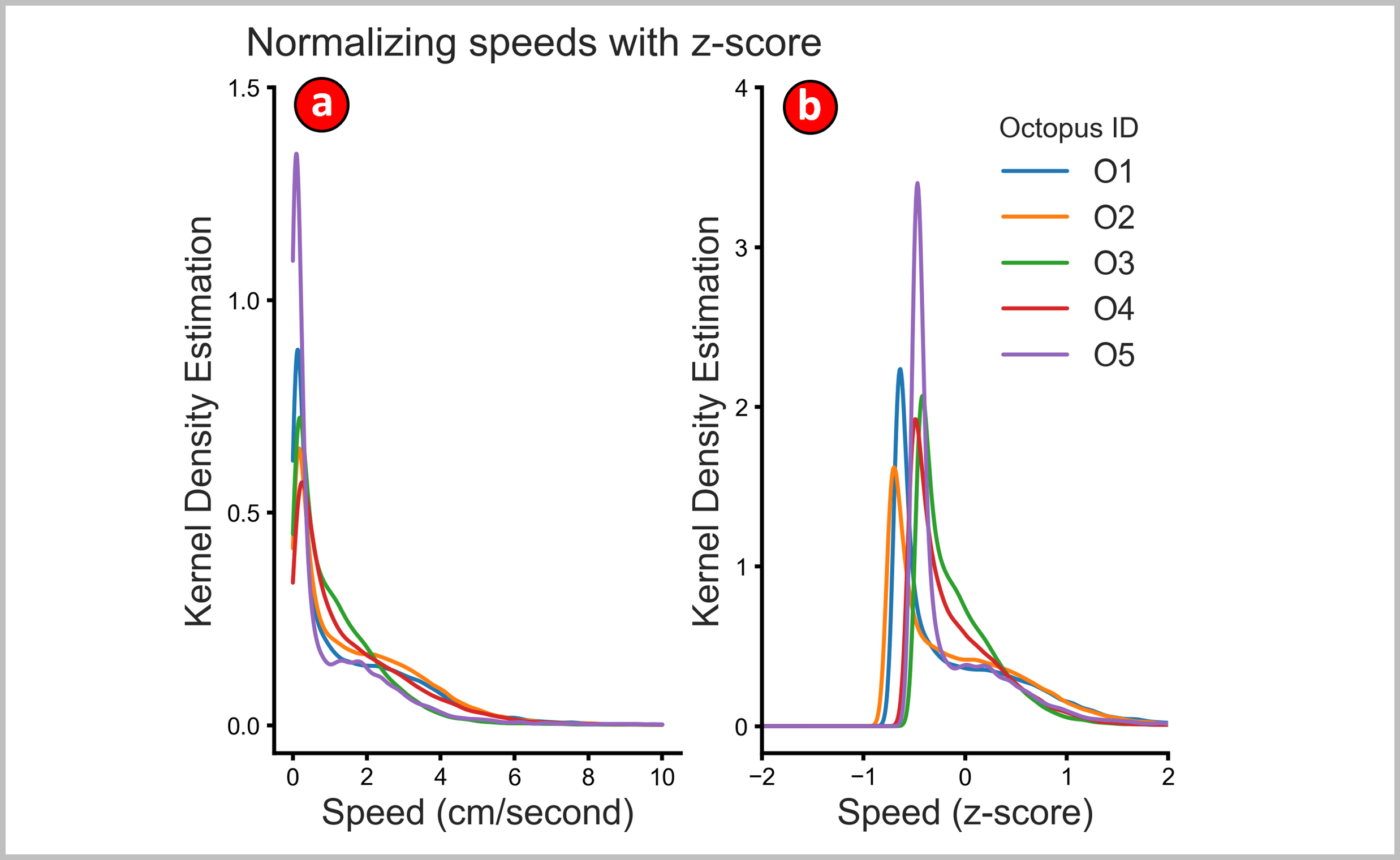

Supplement: S2 Fig — (a) Kernel density estimation (KDE) of the speeds of the five different octopuses used in the single station approach task. The kernel density estimates were calculated after smoothing the velocity traces with a Savitzky-Golay filter. (b) Kernel density estimation of octopus speed z-scores Both (a) and (b) used a KDE bandwidth of 0.05. (TIF) [file pone.0330262.s002.tif]

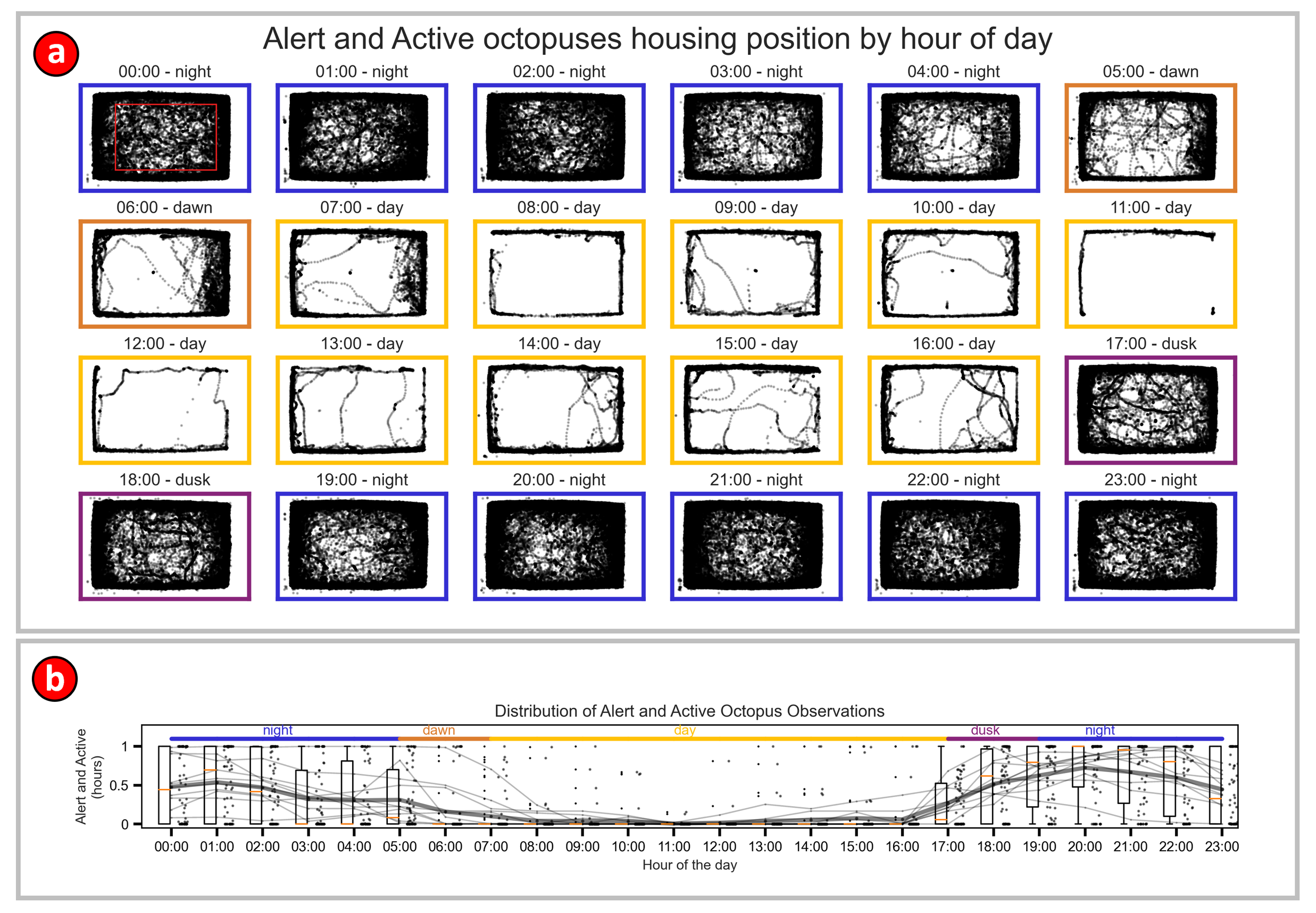

Supplement: S3 Fig — Location of 10 different alert and active octopus, held in the flume at different times, continuously recorded at 10 Hz for a total of 66.9 days of observations. A pair of experimenters exhaustively annotated when the octopus were visibly alert and active in all days of recording. The large size of the flume and relatively low-resolution of the footage made many subtle behaviors unobservable. Consequently, octopus that were stationary for long periods, regardless of quiescent state, were annotated as not alert and not active. (a) Raw unfiltered DeepLabCut mean eye locations of the octopus split by hour of the day Plot boundary colors indicate night, dawn, day, and dusk. (b) Fraction of the time each hour that the octopuses were alert and active The data points are the averages for each octopus obtained after averaging over the entire video dataset. The thin curves show the variation of the averages for each individual octopus across the day. The thick line is the data averaged over all octopuses. (TIF) [file pone.0330262.s003.tif]

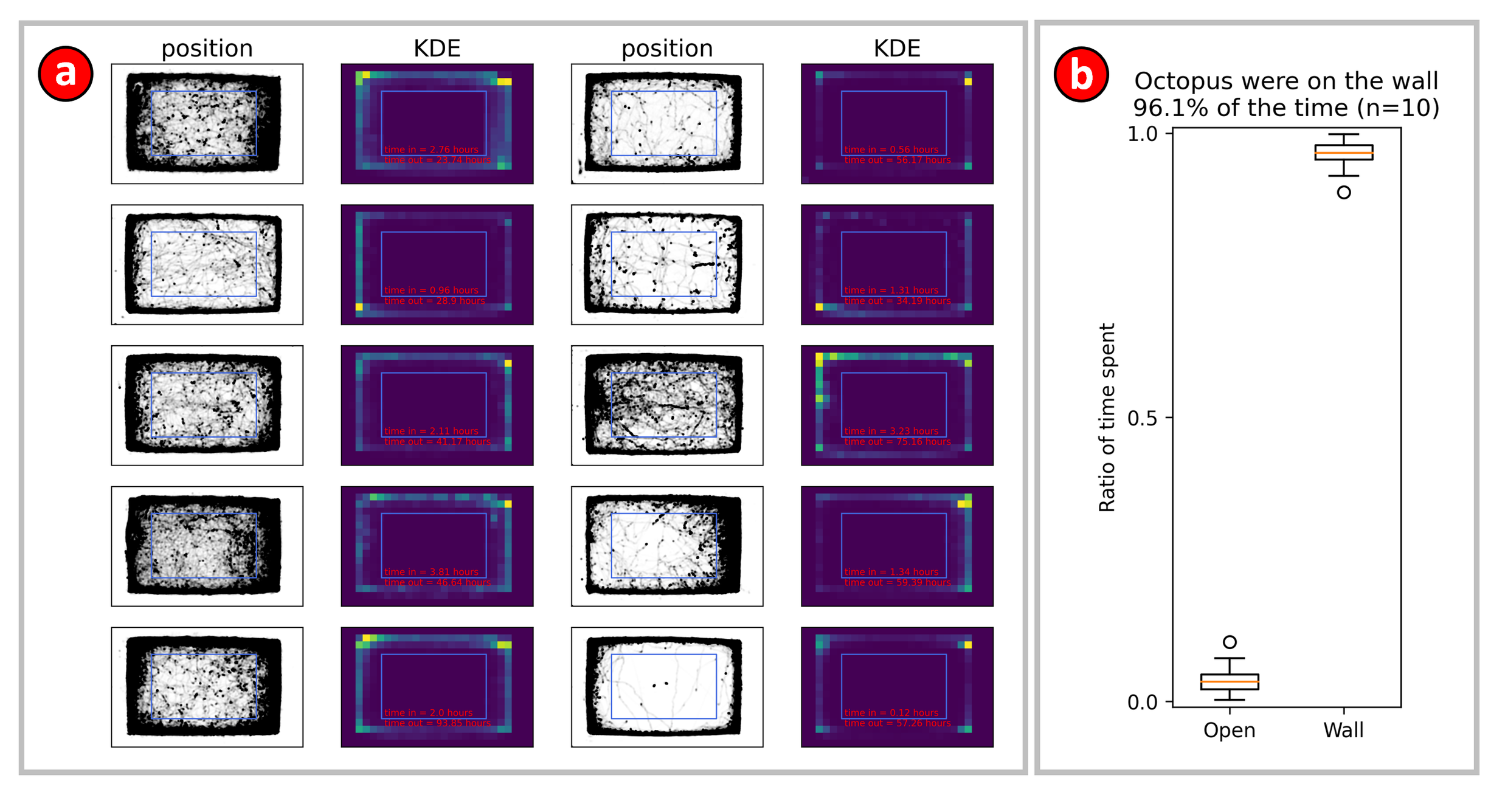

Supplement: S4 Fig — (a) Raw unfiltered DeepLabCut predictions of mean eye location for ten different alert and active octopus in the flume (S3 Fig.). Two-dimensional kernel density estimation (KDE) was used to show the distribution of space occupancy. A blue rectangle within each KDE plot delimits the on and off the wall boundary. Amount of in and out time measured for each individual is shown in red text. (b) Averages of time spent in the open and on the wall by each octopus. On average the octopus spent 96.1% of their time on the wall while alert and active. (TIF) [file pone.0330262.s004.tif]

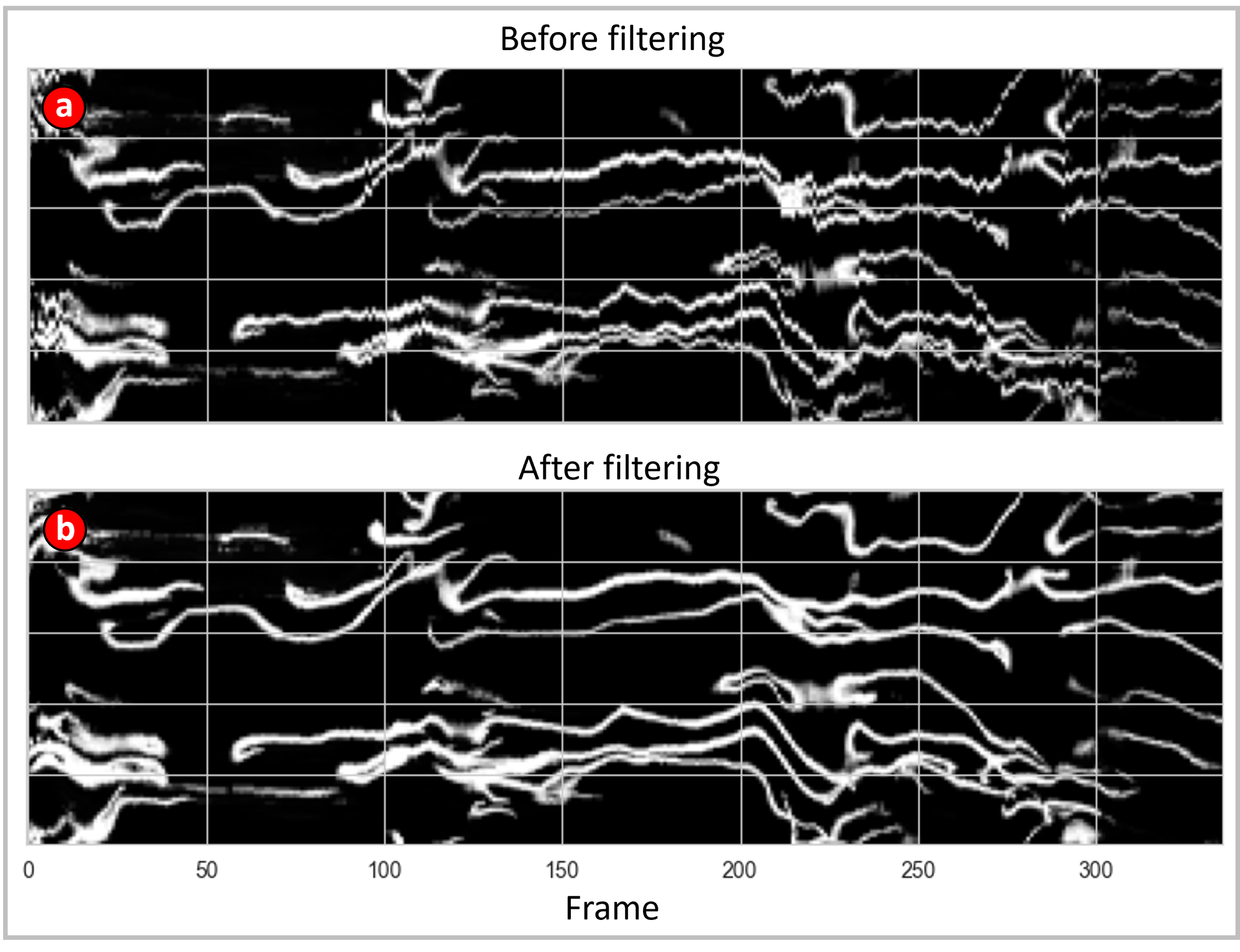

Supplement: S5 Fig — (a) Trace before Savitzky-Golay (SavGol) filtering of the DeepLabCut eye coordinate predictions. Due to the large radius of the bounding circle used to define arm positions (see Fig. 3d), small oscillations due to prediction error in the eye oordinates result in large, spurious fluctuations of the arm positions. (b) Trace after Savgol filtering. High frequency oscillations are noticeably reduced. (TIF) [file pone.0330262.s005.tif]

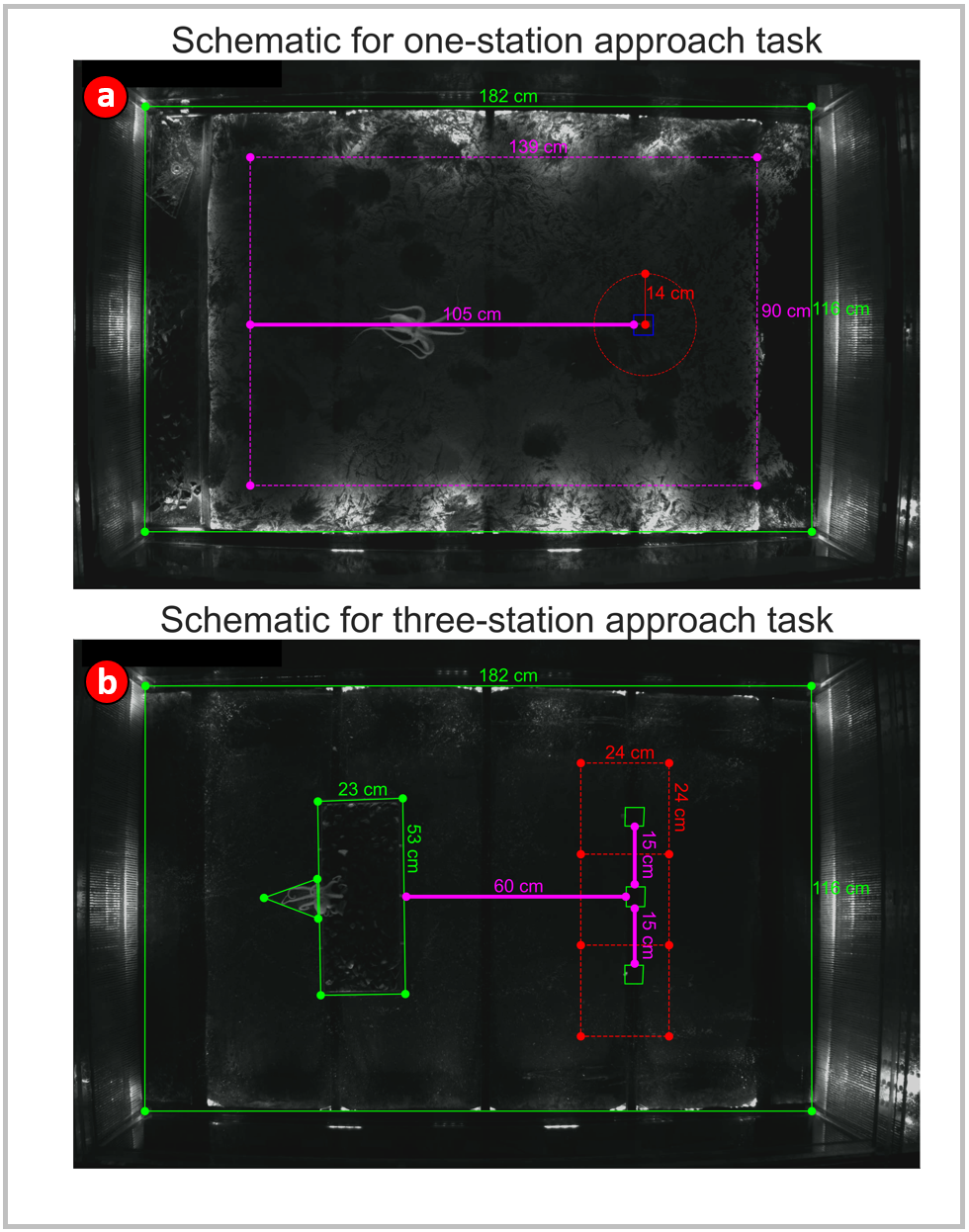

Supplement: S6 Fig — (a) One-station approach task schematic. Dashed magenta rectangle is the bounds of segmented trajectories for the task. Solid magenta line shows the length from the downstream edge of the rectangle to the food station. The red circle segmented the end trajectories that ended with feeding, the circle’s radius is roughly the length of the octopus arms. (b) Three-station discrimination task. Dashed red squares are the segmentation edges of the three station task stations. Small green squares are the location of the stations. Long magenta line is the distance from the downstream rock tray to the stations. Small magenta lines is the distance between the stations. Inner green rectangle and triangle are the rock tray and den respectively. In both (a) and (b) the outer green rectangle visualizes the dimensions of the arena. (TIF) [file pone.0330262.s006.tif]

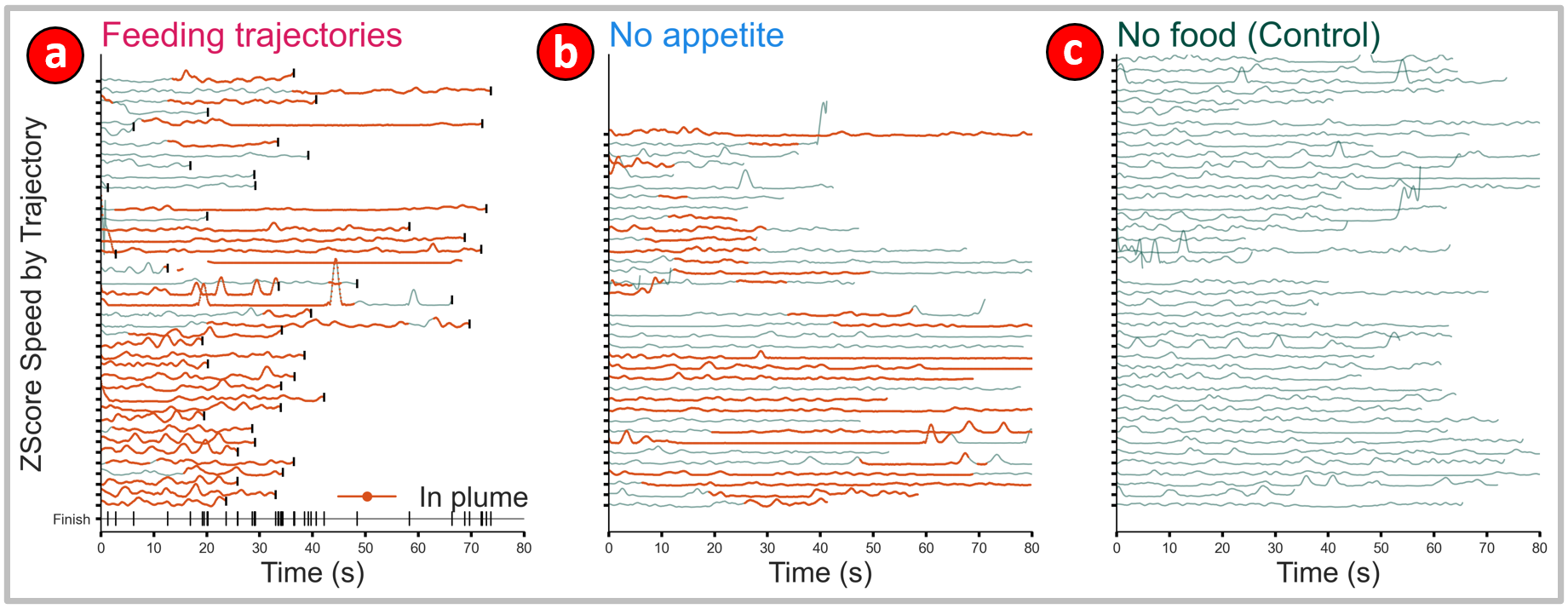

Supplement: S7 Fig — In-plume periods are indicated by orange highlights. (a) All feeding approach trajectories for the single station experiment. Black hatches indicate the moment when the eyes of the octopus crossed into the circle before feeding. (b) All ‘no appetite’ trajectories where food was present but the octopus did not eat. (c) A representative sample of control trajectories in which no food was present. All panels (a-c) were designed with the same vertical and horizontal extents so that direct comparisons could be made by eye. (TIF) [file pone.0330262.s007.tif]
