# Supplementary material for: Octopus track chemosensory plumes to find food
Source: PLoS One. 2025 Oct 8;20(10):e0330262. doi: 10.1371/journal.pone.0330262 (PMC12507230; doi:10.1371/journal.pone.0330262)
Supplement: Attachment — Submitted filename: Response to Reviewers.pdf [file pone.0330262.s008.pdf]

Response to Reviewers

Manuscript Title: Octopus can use odor plumes to find food

Dear Editor,

We thank the reviewers for their thoughtful and constructive comments on our manuscript. We have carefully addressed each point raised, and we believe the manuscript has been significantly improved as a result. Below, we provide detailed responses to each reviewer's comments.

Reviewer #1

The paper titled "Octopus can use odor plumes to find food" is an interesting paper showing that octopuses can perform chemical-plume-guided navigation, a behavior previously known in many animals but never before observed in octopuses. The study shows that octopuses can use "odor" plumes to track and find food, demonstrating that this behavior is not limited to animals with more typical olfactory organs but also occurs in cephalopods like octopuses. While the methods used as well as the collected results are nice and intriguing, the authors should take into account some important works that they ignored.

To avoid further bias in the literature regarding chemoreception, the authors must change the word "odor" to chemical or chemical clue throughout their paper. To understand why, they should carefully read and quote:

- Taste and Smell: a unifying chemosensory theory. by Mollo et al., 2022 `https://doi.org/10.1086/720097`;
- Sensing marine biomolecules: smell, taste, and the evolutionary transition from aquatic to terrestrial life. by Mollo et al., 2014 doi:10.3389/fchem.2014.00092;
- Taste and smell in aquatic and terrestrial environments. by Mollo et al., 2017 DOI: 10.1039/c7np00008a;
- Cephalopod Olfaction. by Di Cosmo and Polese 2017 DOI: 10.1093/acrefore/9780190264086.013.185.

>>> We deeply appreciate the extremely important comment by the reviewer. We completely agree that a unified language is needed for chemosensation and that the binary classification of taste and smell as separate sensory modalities is both incorrect and causes confusion. Hence we have extensively rewritten our paper to the recommendations set forward in Mollo 2022 and Mollo 2017. All the additional citations provided by the reviewer have been added to our text.

Furthermore, for the same reason on why they must use chemicals instead of odor, it is essential that the "odor" or better the chemical clues (molecules) used in the experiment must be clearly specified in the material and methods section. The discussion must also be reformulated in an evolutionary key accordingly.

>>> Small pieces of fresh shrimp were used as the food reward given to the octopus in the behavioral experiments. The complete chemical composition of the shrimp is much too complex for us to quantify fully, and so a complete list of the molecules present in the chemosensory plume emanating from the food source cannot be fully listed.

I believe that the suggested changes will give a pivotal new and more interesting vision of the results obtained that will go further the already incredible technical work done.

As it is now the paper will contribute to confusion in the chemical perception literature and so it must be revised before publication

Reviewer #2

This study by Gire and colleagues is a wonderful and elegant demonstration of olfactory behavior in the octopus. They provide rigorous evidence that octopuses do indeed follow odor plumes, and quantify the behavioral motifs that the animals use to achieve this, including active sensing. The study thereby provides a strong experimental foundation for a phenomenon that had largely been described anecdotally. In addition to the new insight it provides into olfactory processing across the animal kingdom, this will provide a basis for the study of the underlying neural mechanisms in octopus.

I have only a few minor points for presentation.

1. There is no information on the time required to reach the target, which would be useful to get a sense of how efficient the animals are.

>>> We have added a new supplemental figure (Sup. Fig. 7) which shows the time against z-scored speed of the octopus under the different conditions of the single station approach task.

2. I am not sure the figures based on previous anecdotal evidence (Fig 1A-C) are useful here, except to provide contrast with the rigor of the current study, as they aren't really data per se.

>>> We have chosen to retain this figure for the following two reasons. (1) We think that the images provide valuable context to the non-expert reader. (2) These images are also valuable anecdotal evidence and have motivated our study.

3. It would be helpful to have a schematic for the two behavioral setups, including length scale.

>>> We have added a new supplemental figure (Sup. Fig. 6) which has the relevant length scales for both behavioral setups.

4. I wasn't sure what the "sigma" symbol on Fig 4 signifies.

>>> Sigma denotes the sum of the proportional edges of the target boxes. We have rewritten the equation in subfigure 4(d) by adding limits to the sigma summation symbol to help the reader.

5. Fonts in many figures are almost too small to see.

>>> Our figures are designed to be viewed digitally, which allows the reader to zoom in on smaller features. We tried to increase font sizes in the figures, but found that visual clarity was reduced. However, we have magnified the legends in two subfigures (7g, 7j).

Reviewer #3

This is a nicely written report of a very interesting and well designed study. The analysis is impressive and should be of use to other groups working on non-traditional models. Overall I think the study is novel,

valuable and interesting and certainly worth publishing. Whilst I have a number of suggestions, they are all minor.

Introduction: "Efficient use of chemical cues for search and way-finding is an evolutionary pressure experienced by most organisms". Most organisms are not animals, so this should be rephrased

>>> This change has been made. The word 'animals' has replaced 'organisms'.

Figure 1 d: a clearer description of what the diagram here shows would be helpful; first, that this represents a model or a hypothesis, not actual data (at least I don't think it shows actual data, either way this should be clarified), and second, that the fine black lines represent a theoretical odor plume and the red line represents the animals' proposed movement under the three different strategies.

>>> We have added a title to Figure 1d and a sentence to the end of the caption to make it clear that the figure does not represent actual data, but is a schematic representation of three different behavioral chemosensory tracking strategies.

Introduction: "Some mollusks use chemoreception in predator avoidance, although this has yet to be demonstrated for octopuses. For example, both scallops (Speiser and Wilkens, 2016) and gastropods (See Edgell (2010) and references therein) increase escape responses in the presence of chemicals associated with nearby predator activity." The authors may wish to add Howard et al., 2019 here, which demonstrates suppression of feeding activity in the presence of olfactory predator cues in squid.

>>> We have added the Howard et al., 2019 citation.

In the introduction the authors state that chemosensing in odor plumes has been studied in nautiluses only, which is not entirely correct. While they cite Chase and Wells, the clearest example of probable odor-gated rheotaxis in octopus, it is erroneously characterized as a study of chemotactile sensing. Notably, the results in this older paper report "arm waving" behavior, precluding the sensing only of substrate-bound molecules, and what would seem to be the same behavior as the FAAM movement describe here. "Other arm movements were more directed; these were extensions of the arms, which often preceded a locomotion oriented primarily upstream.

>>> We thank the reviewer for pointing out this error. It is indeed correct that Chase and Wells is a study of octopus chemosensory behavior and not just chemotactile. We have changed the language in our introduction. We found a similar passage in our conclusion and have changed the language there as well.

Figure 7 is hard to understand. It looks like there are many more data points represented in the 7c (no food) panel, which makes any inference about differing trajectories very hard to interpret. I assume this is related to the plotting of positions every 2 seconds (although this is stated in the caption for Figure 6 and not figure 7, I assume it is the same), which would then imply that the octopuses moved more slowly in the absence of a directional odor cue. This makes sense but if the point of the figure is to show the more aligned trajectories (i.e., lower heading angles) in the presence of food, this really cannot be discerned from panels a-c.

>>> Figure 7 does not contain statistical analysis, instead it helps the reader visualize the distribution of data points so that they can understand the construction of figure 8. We apologize that Fig 7c is difficult to read but we retain the visual complexity to show the reader the distribution of the data points, which is crucial for understanding figure 8.

>>> We thank the reviewer for highlighting the plotting interval of the hairs. We reviewed our code and found that the interval for plotting was actually three seconds and have made the correction to the language in captions of figure 6 and figure 7.

Figure 8d. There are three different colors shown in the individual images; red arrows but also green and magenta arrows. An explanation for these colors should be given in the figure caption.

>>> We thank the reviewer for pointing this out. The colors are now explained in the caption.

Figure 6 shows an interesting observation that the authors don't really address; from these trajectories it does not appear that there is a significant change in movement speed as the octopuses get closer to the target. Change in velocity is a hallmark of close-distance olfactory search in many species, and it would be interesting for the authors to consider this variable in their analysis and discussion. Although it is hard to make clear determinations from figure 6 there certainly does not appear to be a consistent switch in strategy close to the target. Given the emphasis throughout the manuscript on the difference between octopuses and other animals where olfactory search is more deeply studied, this point could be elaborated.

>>> We thank the reviewer for this excellent observation. We do not think we collected enough data to properly address this topic.

Discussion and conclusions: the authors somewhat ignore one of the most interesting aspects of octopus neurobiology until the opening section of their conclusion, which is the possibility of central processing of odorant information that is received by two different arms. While their point that understanding single-sucker processing is a necessary precursor to building up a model of whole-arm or whole-body processing is fair, both the experiment design and the analysis pipeline really focus on whole-body aspects of tracking behavior, with little emphasis in the analysis of looking at single-sucker behavior. It is known that the olfactory lobes receive inputs and send outputs to the basal lobes where pathways from the arms are found, so it is worth discussing this aspect of octopus neurobiology. The behaviors they describe also raise the intriguing possibility of a somatotopic map of the arms informing the directional search behavior; the apparent absence of central brain somatotopy in cephalopods is of great interest to comparative neurobiologists, as is the apparent absence of glomeruli-like structures in the olfactory lobe itself. A brief discussion of central brain pathways and implications for central processing would broaden the general appeal of the paper. A large section of the discussion is devoted to an explicit comparison between rodents and octopuses, which is perhaps not the most informative comparison.

>>> The reviewer brings up an excellent point. Octopus central processing and/or sharing of chemosensory information between arms is a technically hard problem to address. Statistically significant results for multi-arm tracking is challenging, and with eight arms one would need a dataset much larger than the one we have at present. We believe that progress in understanding the distributed chemosense at the sucker level is key to understanding how the brain is involved. At present it would be fair and probably obvious to say that the brain is likely involved somehow but we cannot say much more than that at the present state of the field.

>>> We added text to the discussion which discusses Messenger and Young's investigation of the olfactory organs and the connections to the basal lobes.

>>> We agree it is possible that octopus have some level of sensory somatotopy of their arms. But we

disagree that the behaviors we describe uniquely point to the existence of a somatotopic map as behavior alone is insufficient to make this claim.

>>> We feel that a discussion of the central chemosensory neural pathways of octopus with other animals is too long for our paper, and not of much value as many comparisons would be quite speculative.

>>> We compared the octopus with rodents because we have expertise with them as a chemosensory model, and we wished to make the point that the octopus chemosensory system seems very different from vertebrates.
